# Effects of Isothermal Treatment on A_g_ZIF‐62: Implications on Porosity, Separations, and Grain Boundary Defect Removal

**DOI:** 10.1002/smsc.202500288

**Published:** 2026-02-12

**Authors:** Dana M. Stone, Cara M. Doherty, Durga P. Acharya, Sarah E. Morgan, Mai O. Abdelmigeed, Jimmy Nguyen, Nathan C. Harvey‐Reid, Elnaz Jangodaz, Shane G. Telfer, Gregory N. Parsons, Matthew G. Cowan

**Affiliations:** ^1^ Department of Chemical and Process Engineering MacDiarmid Institute for Advanced Materials and Nanotechnology University of Canterbury Christchurch 8140 New Zealand; ^2^ MacDiarmid Institute for Advanced Materials and Nanotechnology School of Physical and Chemical Sciences University of Canterbury Private Bag 4800 Christchurch 8140 New Zealand; ^3^ Future Industries Commonwealth Scientific and Industrial Research Organisation Private Bag 10, Clayton South Victoria 3169 Australia; ^4^ Department of Chemical and Biomolecular Engineering North Carolina State University Raleigh NC 27606 USA; ^5^ School of Natural Sciences Massey University Palmerston North 4410 New Zealand

**Keywords:** amorphous materials, melting, metal organic frameworks, porosity, ZIF–62

## Abstract

Glasses derived from metal‐organic frameworks (MOFs) combine the processing benefits of glassy materials with the accessible and selective porosity of MOFs, with potential applications in gas separation and electronics. Establishing control over MOF glasses requires an accurate understanding of how processing parameters will affect the resulting glass properties. To advance this understanding, the effect of isothermal melt treatment conditions on the porosity and morphology of ZIF–62 is investigated. It is demonstrated that the transition from crystal to glass increased in fractional free volume (3.78 ± 0.07% to 5.50 ± 0.03%, respectively) while impeding the accessibility of CO_2_, N_2_, and propene by 59–79%. It is demonstrated that the change in pore volume is independent of isothermal hold times. In contrast, isothermal hold time allows control over glass morphology, where short treatments retained more original morphological characteristics, while longer treatments improved grain coalescence.

## Graphical abstract

This study determines the effects of melt conditions and time on a_g_ZIF‐62's pore volume, accessible porosity, morphology, and coalescence for controlled production of ZIF‐62 glasses.

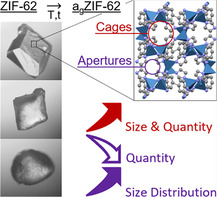

## 1 Introduction

Zeolitic imidazolate frameworks (ZIFs) are a subset of the widely researched porous metal‐organic frameworks (MOFs). ZIFs are crystalline structures comprised of metal M^2+^ ions tetrahedrally bound to imidazolate ligands, which form repeating frameworks of interconnected cages.^[^ ^1^ ^]^ Narrow apertures form passages between these cages, creating an ideal material for separation processes such as molecular sieving,^[^ ^2^ ^]^ adsorption,^[^ ^3^ ^]^ and carbon capture.^[^ ^4^, ^5^ ^]^ Additionally, the wide range of metal ions and imidazolate ligands allows these structures to be tuned for different properties including aperture size, porosity, and thermal stability.^[^ ^6^, ^7^, ^8^, ^9^ ^]^

Processing MOFs into applications that utilize their idealized properties can be difficult due to their crystalline nature. However, the thermal stability of some ZIFs allows the formation of glasses and therefore opens new processing possibilities.^[^ ^1^, ^10^ ^]^ Meltable ZIFs form a highly viscous liquid prior to reaching decomposition temperatures and, upon cooling, retain the liquid‐like amorphous structure of glass.^[^ ^11^ ^]^ Of meltable ZIFs, the amorphous glass (a_g_) form of ZIF–62 is one of the most‐widely researched, due in part to the high ratio between its glass transition temperature (*T*_g_, 330 °C) and melting point (*T*_m_, 410 °C) which impedes recrystallization during the cooling process.^[^ ^12^, ^13^, ^14^ ^]^ a_g_ ZIF–62 has been successfully processed into many different forms, including pellets, foams,^[^ ^15^, ^16^ ^]^ thin films,^[^ ^17^ ^]^ and membranes.^[^ ^18^ ^]^

In addition to processability, ZIF glasses possess niche benefits over crystalline MOFs. Glasses are more rigid,^[^ ^19^ ^]^ which posits that the reduced flexibility on the atomic scale could result in improved molecular sieving.^[^ ^6^ ^]^ ZIF glasses also exhibit altered optical properties, including luminescence^[^ ^20^ ^]^ and, due to their amorphous nature, possess optical isotropy.^[^ ^21^ ^]^ Glasses also exhibit morphological benefits, including the removal of interfacial grain boundary defects in thin‐films,^[^ ^22^, ^23^, ^24^, ^25^ ^]^ improving ion conduction,^[^ ^26^ ^]^ surface passivation,^[^ ^27^ ^]^ and strengthening the glass for more versatile use, such as greater pressure differentials as required in gas separations,^[^ ^17^, ^22^, ^23^, ^24^, ^25^ ^]^

To optimize desirable properties, consideration of how thermal processing affects the structure of the ZIF is required. Most important for applications is the retention of the porous structure in the resultant amorphous glasses, retaining the benefits of crystalline MOFs.^[^ ^28^ ^]^ For example, a_g_ZIF–62 maintains much of the short‐range order of its crystalline precursor, and some porosity is preserved.^[^ ^18^, ^19^, ^29^ ^]^ During this process, the restrictive apertures have been shown to expand by 0.05–0.15 nm by either the formation of new pore structures or the rupture of bonds.^[^ ^8^, ^18^, ^29^ ^]^

Similarly, understanding how melt conditions can be manipulated provides further control over the production of porous glasses. Most relevant to this work, the melt properties of ZIF–62 glasses can be controlled via the ligand ratio of imidazolate and benzimidazolate,^[^ ^8^, ^11^, ^30^ ^]^ where additional benzimidazolate increases *T*_m_ and *T*_g_.^[^ ^8^, ^11^, ^21^, ^30^ ^]^

Likewise, providing inert atmospheric conditions during heating prevents thermal decomposition such as in Ar, which increased ZIF–62's decomposition temperature (*T*_d_) by 160 °C giving a greater window between *T*_m_ and *T*_d_ for glass production.^[^ ^21^, ^31^ ^]^

Melt temperature, time, and annealing conditions^[^ ^32^ ^]^ are also correlated to the glass properties, such as the decrease of *T*_g_ and pore size attributed to changes in medium‐range order.^[^ ^21^, ^33^ ^]^ Effects include density, hardness, elastic moduli, morphology (foam vs. coherent glass).^[^ ^34^, ^35^ ^]^ The above considerations illustrate the importance of understanding how precise material selection and tailoring can control properties and unwanted variations.

Having highlighted the importance of thermal behavior for controlling glass properties of a_g_ZIF–62, it is equally important to draw attention to the variation reported in literature for a_g_ZIF–62 production. A comprehensive summary of the thermal treatments on ZIF–62 showed differing heating rates, temperatures, isothermal stage duration, environments, and heat sources across literature (Figure S2 and Table S2, Supporting Information). Each inconsistency causes problems when comparing outcomes of different papers, be it a_g_ZIF–62 properties or applications. Consideration must also be given to the method of heating. For instance, tubular furnaces (prevalent equipment used for melting ZIF–62) can lack precision in temperature control (Figure S3, Supporting Information), exacerbated by differences in sample location, furnace size, heating rates and gas flow rates.^[^ ^36^, ^37^ ^]^

To resolve these inconsistencies and provide a systematic understanding of how melt conditions can control the porosity and morphology of a_g_ZIF–62, this work systematically assesses the contribution of isothermal duration at melting and annealing temperatures during a_g_ZIF–62 processing.

## 2 Results and Discussion

The ZIF–62 glasses assessed within this work were produced via differing isothermal treatment durations at *T*_m_ (410 °C) and *T*_g_ (330 °C) and are henceforth transcribed by the times maintained at each temperature ‘(*t*_m_, *t*_g_)’, e.g., (0, 300) indicating heating to *T*_m_ with no isothermal treatment time, followed by isothermal treatment of 300 min at *T*_g_. To eliminate the possibility of destructive combustion causing variations in porosity and morphology, retention of mass during treatments was confirmed using TGA analysis (Table S3, Figure S5, Supporting Information).^[^ ^38^ ^]^ Powder X‐Ray diffraction confirmed the complete amorphization of a_g_ZIF–62 (Figure S4, Supporting Information). Full experimental procedures are provided in Section 1 of the Supplementary information.

### 2.1 Porosity

**Figure 1:**
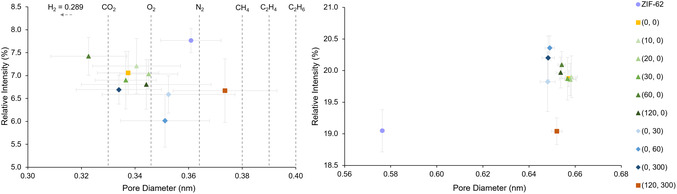
The intensity (relative number) and diameter of the pores apertures (left) and pore cages (right) in ZIF‐62 and a_g_ZIF‐62 variants (denoted by (*t*_m_, *t*_g_) in min) found via a 4‐component fit (two pore model) from PALS. The kinetic diameter for various gases are added as comparison to the pore apertures.

**Figure 2:**
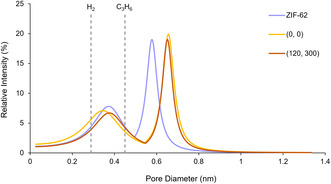
PSDs of ZIF‐62, and a_g_ZIF‐62 melted at the extremes of isothermal hold times fit from PALS. The kinetic diameter of H_2_ and C_3_H_6_ have been added for comparison.

The framework aperture is linked to molecular sieving capability and therefore a key variable in gas separation applications. PALS characterization of ZIF–62 (**Figure** **1**) revealed pore diameters of 0.36 ± 0.01 nm, consistent with previous work,^[^ ^8^, ^18^ ^]^ which underwent a negligible decrease of –0.02 ± 0.01 nm following melting, even with longer isothermal durations. However, the range of pore diameters across a_g_ZIF–62 was large (0.32 ± 0.01 to 0.37 ± 0.02 nm). The variation between glass pore apertures has problematic implications for gas separation performance because the kinetic diameter of CO_2_, O_2_, N_2_, and CH_4_, all fall within this range. This variation is consistent across all glass samples, indicating an unpredictability for a_g_ZIF–62 production. Furthermore, our results indicate that this variation cannot be controlled or reduced by annealing treatments (Figure 1). The pore size distributions (PSDs) have further implications for molecular sieving capabilities. The range of apertures seen in both ZIF–62 and a_g_ZIF–62 within individual samples extends above the kinetic diameter of propene (0.45 nm) (**Figure** **2**). While the quantity of these larger apertures is low, they could reduce the selective properties of the framework.

PALS revealed significant differences between crystalline and glass forms. The intensity, i.e., relative number of apertures, decreased by 12 ± 4% upon melting, whereas the framework cages increased in both size (from 0.576 to 0.654 ± 0.001 nm) and intensity (from 19.1 ± 0.3 to 19.9 ± 0.1%) for ZIF‐62 and a_g_ZIF–62 respectively. These experimental results match predictions from a nonlocal density functional theory by Frentzel‐Beyme et al.,^[^ ^8^ ^]^ who identified a “collapse” (loss) of apertures and ≈1/2 the cages contrasted by the formation of larger pores (0.8 nm diameter) in a_g_ZIF–62. An alternate visualization of this process that links the contrasting aperture and cage intensity observations is the disruption of the aperture bonds during thermal treatment eventually leading to bond dissociation and the merging of adjoining cages.

In addition to aperture and cage sizing, porosity has a considerable impact on potential applications, particularly gas separation. In alignment with previous characterization using PALS,^[^ ^11^ ^]^ the fractional free volume increased significantly between ZIF–62 and the a_g_ZIF–62 variants (3.78 ± 0.07% and 5.50 ± 0.03%, respectively). For membrane‐based separations, high porosities result in greater permeance, provided the cavities are interconnected.^[^ ^39^ ^]^ For adsorption‐based separations or host‐guest property optimization, higher porosity is indicative of a higher loading capacity.^[^ ^40^, ^41^ ^]^ In previous studies, BJH modelling of adsorption isotherms suggested that 60–64% of the accessible pore volume to N_2_ was lost following thermal treatments.^[^ ^17^ ^]^ The PALS results, which consider all pore volume, including unconnected pores, reported herein suggest that an overall increase in pore volume actually occurs during melting to form a_g_ZIF‐–62. A likely explanation is that the collapse of 12 ± 4% of the apertures prevents N_2_ from accessing the newly created pore volume due to poor interconnectivity. This highlights the usefulness of BJH for measuring pore volume physically accessible to N_2_, while cautiously noting the sensitivity of BJH to changes in aperture size of a_g_ZIF–62.

To identify if smaller gases had greater access to the pore volume, the adsorption of CO_2_, N_2_, and propene were measured at 20 °C within ZIF–62 (30.3 cc_CO2_ g^−1^; 4.4 cc_N2_ g^−1^; 33.8 cc_C3H6_ g^−1^), a_g_ZIF–62 (0, 0) (12.4 cc_CO2_ g^−1^; 0.9 cc_N2_ g^−1^; 8.2 cc_C3H6_ g^−1^), and a_g_ZIF–62 (120, 300) (12.1 cc_CO2_ g^−1^; 1.0 cc_N2_ g^−1^; 8.1 cc_C3H6_ g^−1^) (Figure S6, Supporting Information).^[^ ^42^ ^]^ Melting causes a drop in adsorption capacity for all gases; however, a_g_–ZIF62 retained the highest capacity for the smallest gas CO_2_ (41% retention) compared to N_2_ (21% retention) and propene (24% retention), respectively. Comparing the gas adsorption results with PALS indicates that a_g_–ZIF62 retains porosity that is partially inaccessible to gas molecules, particularly larger gases, resulting in a material with greater selective properties, though at the cost of adsorption capacity.

As anticipated from the PALS results, annealing time did not influence gas capacity, and the glasses (0, 0) and (120, 300) displayed similar adsorption properties to each other, with minimal differences in loading capacity (2–13%) across all adsorbates (Figure S6, Supporting Information).

### 2.2 Morphological Characteristics

**Figure 3:**
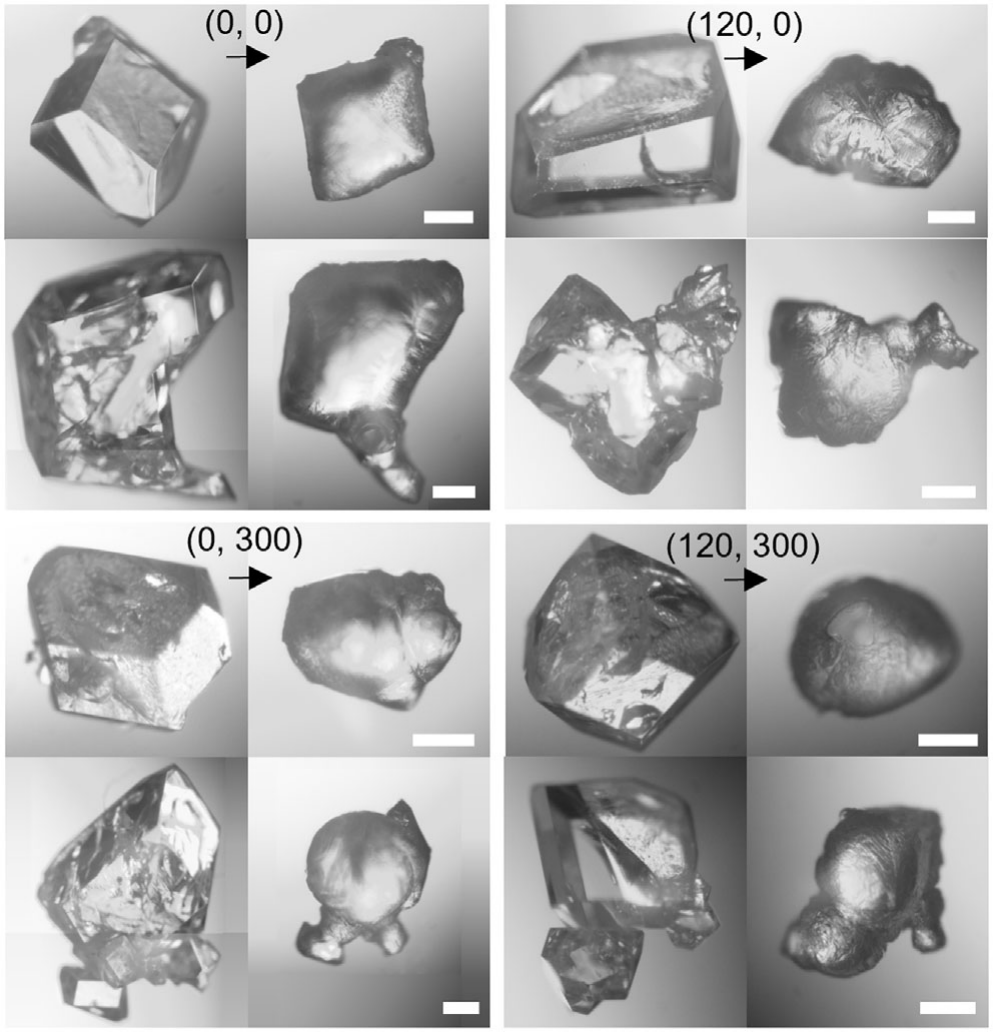
Microscope images of individual ZIF‐62 crystals before and after thermal treatments (0, 0), (120, 0), (0, 300) and (120, 300). Additional images can be seen in Figure S9–12, Supporting Information. Scale bars are 20 μm.

To investigate the contribution of isothermal treatment time to the removal of interfacial grain boundary defects, we tracked the morphologic changes of individual crystals across different isothermal treatments on ZnO surfaces. The a_g_ZIF–62 had low wettability against a ZnO surface during melting, seen in **Figure** **3** and S7–10, Supporting Information, by the formation of almost spherical glass beads and a lack of dispersal along the ZnO surface. The notably greater sphericity in samples treated for longer durations is due to the high viscosity of liquid ZIF–62 (*η* = 10^5.1^ Pa s) and greater time window of fluidic movement.^[^ ^11^ ^]^ These results indicate that shorter melt times are required to retain morphology and/or that optimizing surface wetting will play an important role in the morphology of ZIF glasses.

**Figure 4:**
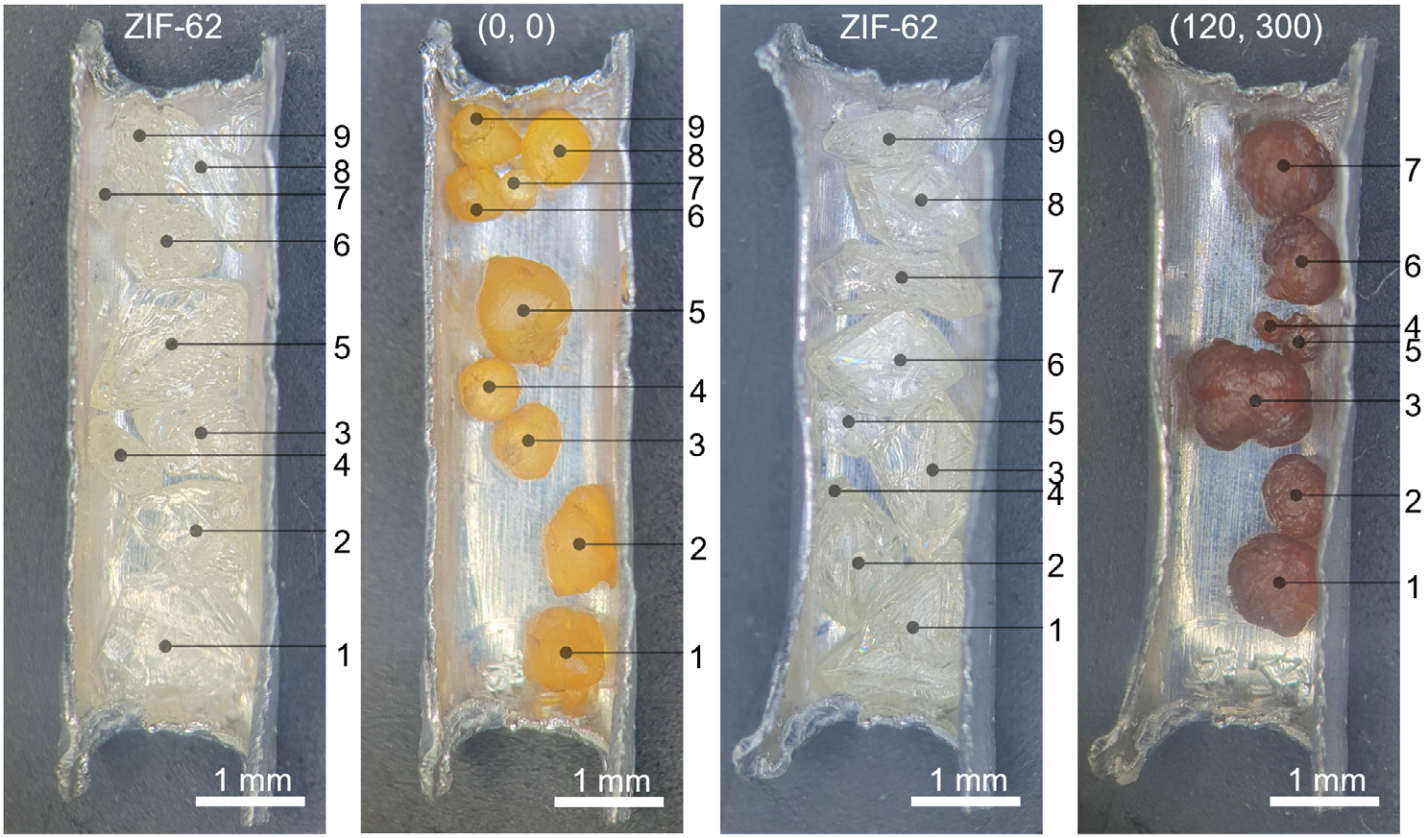
Monolayer arrangement of macroscale ZIF‐62 grains, before and after thermal treatments. Coalescence is seen by the reduction of individual beads following melting.

Importantly, for assessing the ability to heal interfacial defects, samples composed of semiconjoined grains showed retention of their shape at the connection points during melting. Behavior of adjacent grains was assessed to determine the melting conditions required to allow coalescence. Following thermal treatments, paired ZIF–62 grains (sized 0.5–2 mm) only coalesced at (120, 300), whereas the (0, 0) samples remained as distinct beads (Figure S13, Supporting Information). This same trend applied to monolayer arrangements of ZIF–62 grains where coalescence only occurred with long isothermal treatment durations (**Figure** **4**). For these monolayer arrangements, contact between individual grains during the melting phase became more likely with the increased isothermal duration. However, the combination of shrinkage associated with thermal treatment and poor surface wetting driving sphericity meant the grains predominantly retreated from each other.^[^ ^17^, ^43^ ^]^ Therefore, to produce coherent ZIF‐glass films or designed structures, a tight arrangement, controlled surface wetting, or an additional driving force (such as pressure molding) is required to ensure coalescence.

Acknowledging that many applications of ZIF‐glasses will be microscale devices, the effect of macro vs microscale on melt behaviors was examined. ZIF–62 was grown directly onto a 50 nm surface layer of ZnO (deposited via atomic layer deposition) resulting in a monolayer of grains ≈10 μm in diameter (Figure S14, Supporting Information). The closer proximity of the microscale grain resulted in better significantly better coalescence during melting than the macrocrystals, with precursive grains becoming unidentifiable. The eased thermal distribution through the microscale grains, along with preattachment to the surface improved the coalescence to the point where differing isothermal treatments cause little discernable morphological variation between the resulting films.

## 3 Conclusion

This work provides valuable insights to those seeking to produce gas separation and electronics devices from ZIF glasses. The effect of glass formation conditions, i.e., melting and annealing times, was shown to maintain total porosity while slightly constricting apertures and increasing the size of cages. However, gas adsorption studies showed that some of this porosity is no longer accessible, particularly for larger gases N_2_ and propene. These experimental results support past theoretical studies advocating an aperture collapse mechanism during melting. While isothermal treatment times at *T*_m_ and *T*_g_ cause no statistically significant variation in porosity, they do significantly affect the morphology of melted a_g_ZIF–62, especially at the macroscopic scale. In particular, the control of isothermal treatment times can be used to improve crystal grain coalescence and morphology, as required for effective membranes, ion conduction, and surface passivation applications.^[^ ^44^, ^45^, ^46^, ^47^, ^48^, ^49^, ^50^, ^51^, ^52^, ^53^, ^54^, ^55^, ^56^, ^57^, ^58^, ^59^, ^60^, ^61^, ^62^, ^63^, ^64^, ^65^ ^]^

## Supporting Information

Supporting Information is available from the Wiley Online Library or from the author.

## Conflict of Interest

The authors declare no conflict of interest.

## Supporting information

Supplementary Material

## Data Availability

The data that support the findings of this study are available in the supplementary material of this article.
