# Supplementary material for: Effects of Isothermal Treatment on AgZIF‐62: Implications on Porosity, Separations, and Grain Boundary Defect Removal
Source: Small Sci. 2026 Feb 12;6(2):e202500288. doi: 10.1002/smsc.202500288 (PMC12928020; doi:10.1002/smsc.202500288)
Supplement: Supplementary file 1 — Supplementary Material [file SMSC-6-e202500288-s001.pdf]

Supporting Information

Effects of isothermal treatment on a_gZIF-62: implications on porosity, separations, and defect removal

Dana M. Stone,[a] Cara M. Doherty,[b] Durga P. Acharya,[b] Sarah E. Morgan,[c] Mai O. Abdelmigeed,[c] Jimmy Nguyen,[c] Nathan C. Harvey-Reid,[a] Elnaz Jangodaz,[d] Shane G Telfer,[d] Gregory N. Parsons,[c] Matthew G. Cowan*[a]

Abstract: Glass derived from metal-organic frameworks (MOFs) combine the processing benefits of glassy materials with the accessible and selective porosity of MOFs, with potential applications in gas separation and electronics. Establishing control over MOF glasses requires an accurate understanding of how processing parameters will affect the resulting glass properties. To advance this understanding, we investigate the effect of isothermal melt treatment conditions on the porosity and morphology of ZIF-62. We demonstrate that the transition from crystal to glass increased in fractional free volume (3.78 ± 0.07 % to 5.50 ± 0.03 %, respectively) while impeding the accessibility of CO_2, N_2, and propene by 59-79%. We demonstrate that the change in pore volume is independent of isothermal hold times. In contrast, isothermal hold time allows control over glass morphology, where short treatments retained more original morphological characteristics whilst longer treatments improved grain coalescence.

DOI: 10.1002/anie.2021XXXXX

Supporting Information

Table of Contents

Experimental Procedures **3**
Atomic Layer Deposition 3
ZIF-62 Synthesis 3
Thermal treatment 3
Characterisation 4
Results and Discussion **5**
Literature Review of a_gZIF-62 Thermal Treatments 5
PXRD 9
TGA and DSC 10
PALS 12
Adsorption 13
Morphological and Optical Changes 14
^{1}H NMR and infrared characterization 21

Supporting Information

Experimental Procedures

Atomic Layer Deposition

Zinc oxide (ZnO) was applied to the surface of alumina thin layer chromatography sheets (TLCs) and silicon wafers via atomic layer deposition (ALD). Conditions during ALD were taken from previous research.[1] The ALD coating chamber was set to 91 °C and 1.25 Torr. Alternating doses of diethylzinc (DEZ) and water (H_2O) were loaded onto the TLCs separated by purges with N_2. The timing of these doses were 1s DEZ: 30s N_2: 1s H_2O: 30s N_2. Each cycle had a growth rate of 1.8 Å and was repeated until 50 nm of ZnO was deposited. Thickness was confirmed via J.A. Woollam alpha-spectroscopic ellipsometry on silicon wafers.

ZIF-62 Synthesis

ZIF-62 was synthesised in both bulk form and supported on TLCs via a preestablished method.[1] For the bulk, a solution of N,N-dimethylformamide- (DMF), zinc nitrate hexahydrate ($Zn(NO_3)_2{\cdot}6H_2O$) and imidazole (Im) was produced with a molar ratio of 175:1:23.5. To counteract the preferential binding of Zn to benzimidazole (bIm), 20 min of constant agitation were applied to the solution allowing the initial organization of Zn-Im bonds.[2] bIm was then added for a Im:bIm ratio of 10:1. For the supported ZIF-62 synthesis, ZnO was substituted in for $Zn(NO_3)_2{\cdot}6H_2O$. The supports were left in the DMF/Im solution for 20 minutes however without agitation to prevent dislodgment of the ZnO layer from the support surface. bIm was then added in the same ratio as the bulk. The final solutions (either for bulk or with support) were sealed in Teflon lined autoclaves and heated to 130 °C for 48 hr. Once cooled, the ZIF-62 supports were removed from solution and the bulk crystals were filtered from the solution, before being cleaned via five methanol (MeOH) washes to remove excess DMF. Drying occurred over 62 hrs under a 1 Pa active vacuum.

Due to large quantity requirements in downstream testing, multiple syntheses were required which could have caused unreliable data due to batch-to-batch variations in ZIF-62 quality. To ensure consistency all batches were thoroughly combined so if variation occurred between syntheses, they would be present in all samples. A natural settling of smaller crystalline grains towards the bottom of the container also occurred. Requiring agitation of the bulk prior to sample division, along with care to include a representative distribution of grain sizes in each sample

Thermal treatment

Ten variations of a standard melt protocol were followed to access the impact of thermal treatment of the resulting amorphous glass (a_g) form of ZIF-62. This standard protocol involved a two-stage melting process of the ZIF-62. Within a tubular furnace (Furnace GSL-1500X-40, see calibration below Figure S3), a quartz sample boat is loaded into a quartz processing tube, aligning the sample with the centre of the furnace. The processing tube is sealed and flushed with an inert 99.9% N_2 environment (50 mL·min^{-1} flow through). ZIF-62 was heated at 10 °C·min^{-1} to the melting temperature 410 °C (T_m), cooled naturally, reheated to the glass transition temperature 310 °C (T_g), and cooled once more to room temperature (
Figure S1). The thermal treatment variations are referred to by time spent at melt temperature (t_m) and time spent at the glass transition temperature (t_g), formatted as (t_m, t_g), and the full list of conditions tested are displayed in Table S1 below. ZIF-62 itself, which undergoes no thermal procedure is denoted as (-, -).

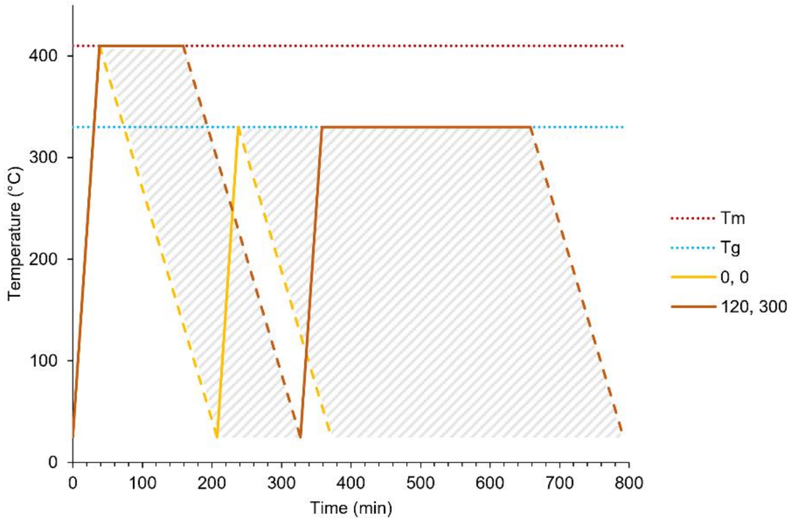

Figure S1. Heating profiles applied to ZIF-62 during the formation of agZIF-62. Variation occurs in the length of time the temperature is maintained at both T_m and T_g, with 0,0 (yellow) representing the shortest length of time, and 120,300 (orange) representing the longest. The region highlighted in grey represents the range within which additional heating profiles were tested, see Table S1.

Supporting Information

Table S1. Variations on the thermal treatments applied to ZIF-62.

Temperature (°C)		Time (min)											
T_m	410 [1]	t_m	-	0	10	20	30	60	120	0	0	0	120
T_g	330 [1]	t_g	-	0	0	0	0	0	0	30	60	300	300

Characterisation

Characterisation of ZIF-62 and a_gZIF-62 was performed on a Rigaku 3kW Smartlab Powder X-ray Diffractometer (PXRD), using Cu Kα radiation and a Cu Kβ filter. 1D data was collected from 3-50° at a 1 °·min^{-1} scan rate with 5° soller slits, and processed on the accompanying Rigaku PDXL2 software. A comparative ZIF-62 simulation was taken from the Crystallographic Open Database.

A Netzsch Jupiter 449 F3 STA was used to perform thermogravimetric analysis (TGA) and Differential scanning calorimetry (DSC) and assess degradation of ZIF-62 throughout the thermal treatment, including varied isothermal stages.10 mg of sample were loaded into Al_2O_3 pre-fired crucibles, and heated following the same protocols outlined above in thermal treatment.

Optical images were taken via a mounted camera on the AmScope T690C Trinocular Compound Microscope, using a 4X magnification lens.

Positron Annihilation Lifetime Spectroscopy (PALS) was undertaken using Ortec EG&G Spectrometers with a timing resolution of 240 ps. The samples were packed around a ^{22}NaCl positron source (50 µCi) sealed in a Mylar envelope. The samples were measured at room temperature under vacuum (1x 10^{-5} torr). A source correction of 1.459 ns and 3.25% was used to account for the mylar. A minimum of five files, each with 4.5 x 10^{6} integrated counts was collected to ensure there was no changes over time. The spectra were analysed using LT-v9 software[3] and fitted to four lifetime components. The first component was fixed to 0.125 ns and attributed to the para-positronium annihilation while the second component ~0.4 ns was due to free annihilation. The third and fourth lifetimes were due to ortho-positronium annihilation. These long-lifetime components were used to calculate the average pore sizes within the samples using the Tao-Eldrup relationship;[4]

$$\tau_3 = \frac{1}{2}\left(1 - \frac{r}{r+\Delta r} + \frac{1}{2\pi}\sin\left[\frac{2\pi r}{r+\Delta r}\right]\right)^{-1}$$

Here, τ_3 is the lifetime (ns), r is the average radius of the pore and Δr is the empirically fitted electron layer thickness (1.66 Å).

Adsorption/desorption isotherms were measured using a volumetric adsorption Quantachrome Autosorb iQ2. High purity gases supplied by BOC Gases were used. Approximately 100-250 mg of the samples were placed in pre-weighed sample tubes. The samples were activated at 150°C for 700 min at a heating rate of 5°C·min^{-1} under a dynamic vacuum. Accurate sample masses were recorded from the activated samples following backfilling with nitrogen. CO_2, N_2 and propene were tested separately at standard temperature/pressure. The 20 °C temperature was controlled by a circulating thermostat-controlled bath filled with a mixture of water and ethylene glycol.

Results and Discussion

Literature Review of a_gZIF-62 Thermal Treatments

A literature review of a_gZIF-62 melting methods was performed to identify what parameters impact the resulting glasses. Huge variability was found in the melting protocols as highlighted in Figure S2 and

Table S2 below. This is concerning when comparing differing literature as even small variances could impact the glasses properties. The impact of isothermal treatments was identified as not systematically investigated within literature and therefore became the key point of research for this work.

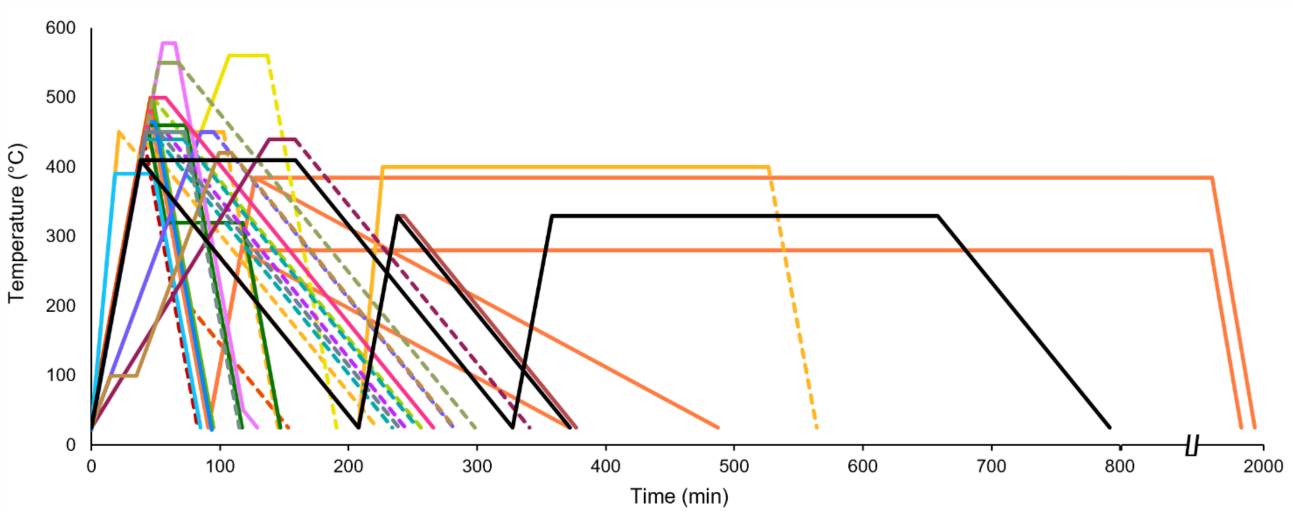

Figure S2. Comparison of temperature profiles used across literature to produce a_gZIF-62 (coloured),[1, 2b, 5] including those used in this work (black). In some cases, multiple profiles were used within a reference, in which case the minimum and maximum profiles (for both temperature and time, as applicable) are displayed. Wherever ramp rate information was unavailable, 10 °C min-1 was assumed, while for natural cooling, a simplified linear cooling rate of 2.3 °C min-1 (based on the average cooling time from 410 to 25 °C min-1 of an available tube furnace); all assumed rates are displayed as dashes. Note the time axis was manipulated above 800 min for readability.

Table S2. Summary of conditions to produce a_gZIF-62 across literature. In addition to the temperature profiles summarised in Figure S2, differences in the heating methods such as heat sources are included here.

Heat Source [A]	Gas	Pressure	T_i	Rate	T_2	Hold	Rate	T_3	Hold	Rate	T_4	Hold	Rate	T_5	Note	Ref
			°C	$°C\cdot min^{-1}$	°C	min	$°C\cdot min^{-1}$	°C	min	$°C\cdot min^{-1}$	°C	min	$°C\cdot min^{-1}$	°C		
TF	Ar	Atm	25	NA	437	0	NA	25	-	-	-	-	-	-		[5e]
DSC	Ar	Atm	40	10	500	0	10	200	0	Nat	25	-	-	-		[5f]
NA	Ar	Atm	25	10	480	0	10	25	0	10	280, 286, 296, 306, 314, 385	0, 10, 30, 60, 240, 720, 1440	1	25		[5g]
TF	Ar	Atm	25	10	437	0	Nat	25	-	-	-	-	-	-		[5h]
HP	Air	Vac	25	NA	450	60	NA	25	-	-	-	-	-	-		"
TF	Ar	Atm	25	20	450	0	Nat	25	0	20	400	300	Nat	25	[B]	[5h, 5i]
HP	Air	Vac	25	5	560	30	NA	25	-	-	-	-	-	-		[5j, 5k]
TF	Ar	Atm	25	10	500	0	Nat	25	-	-	-	-	-	-		[5l]
TF	Ar	Atm	25	10	500	0	10	25	-	-	-	-	-	-		[2b]
TF	N_2	Atm	25	10	460	0, 1, 2, 5, 10, 15, 20, 25, 30	10	320	30	10	25	-	-	-		[5m]
TF	Ar	Atm	25	10	440	10, 30	Nat	25	-	-	-	-	-	-		[5n]
TF	Ar	Atm	25	20	390	30	10	25	-	-	-	-	-	-	[C]	[5o, 5p]
TF	N_2	Atm	22	10	465	5	10	22	-	-	-	-	-	-		[5q]
TF	N_2	Atm	25	5	450	10	Nat	25	-	-	-	-	-	-		[5r]
F	Ar	Atm	25	10	450	15	Nat	25	-	-	-	-	-	-		[5s]
DSC	Ar	Atm	25	10	578	10	10	50	0	Nat	25	-	-	-		[5t]
TF	Ar	Atm	25	10	500	10	Nat	25	-	-	-	-	-	-	[D]	[5u]
NA	Ar	Atm	25	3	440	20	Nat	25	-	-	-	-	-	-		[5v]
TF	N_2	Atm	25	10	410	0	Nat	25	0	10	330	5	Nat	25		[1]
TF	Ar	Atm	25	5	100	20	5	420	10	Nat	25	-	-	-		[5d]
TF	Ar	Atm	25	NA	550	15	Nat	25	-	-	-	-	-	-		[5c]
NA	N2	Atm	25	NA	450	30	NA	25	-	-	-	-	-	-	[E]	[5b]
TF	Ar	Atm	25	10	450	10	Nat	25	-	-	-	-	-	-		[5a]

[A] Acronyms used within the table are as follows; TF = Tubular Furnace, DSC = Differential Scanning Calorimeter, NA = Not Available, HP = Hot Press, F = Furnace, Ar = Argon, N_2 = Nitrogen, Atm = Atmospheric pressure, Vac = Vacuum, Nat = Natural. [B] Ball milled between heating cycles. [C] ZIF-62/6FDA-DAM polymer matrix. [D] ZIF-8/ZIF-62. [E] ZIF-62/ZIF-7

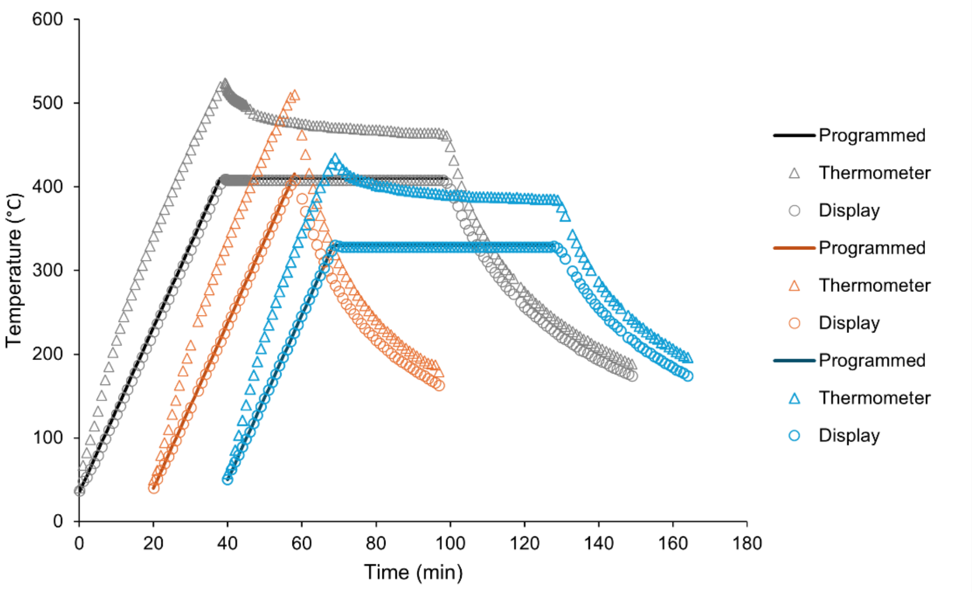

Figure S3. Calibration of a tubular furnace, comparing the programmed temperature profile, displayed temperature determined by the inbuilt furnace sensors, and the actual temperature at the centre of the tube. Due to the overshoot of the true temperature, samples would reach temperatures 25 – 30% greater than intended and be above the intended T_{max} for an additional 15 minutes. Measurements have been offset along the time axis for clarity.

PXRD

Powder X-ray diffraction was used to identify and characterise the ZIF-62 and a_gZIF-62 samples. The ZIF-62 sample was tested and matched to a simulated ZIF-62 pattern, while the a_gZIF-62 samples were tested to confirm full amorphization, seen by the absence of sharp peaks across the diffractogram.

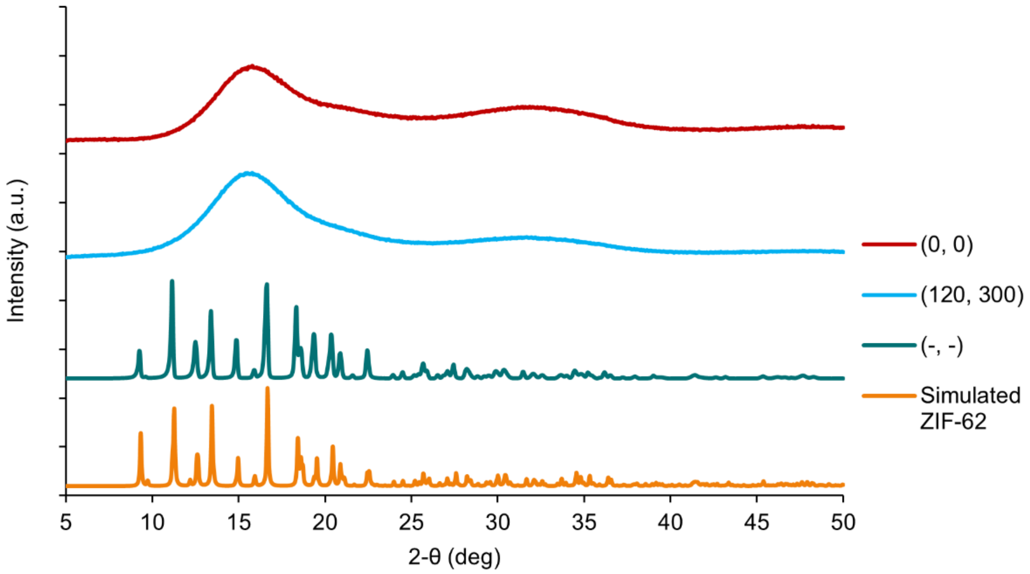

Figure S4. PXRD data of synthesised ZIF-62 (-, -), and glass ZIF-62 following two different melting protocols (0, 0) and (120, 300). Synthesis of ZIF-62 was confirmed against its simulated ZIF-62 pattern.[6]

Supporting Information

TGA and DSC

Thermogravimetric analysis was used to assess whether mass was being lost during the thermal treatments, particularly whether the isothermal holds would cause unwanted mass losses associated with decomposition. The most significant losses in all temperature profile tests were at 153 °C due to the release of the trapped solvent DMF. While the framework maintained a steady mass throughout the rest of the heating and isothermal holds.

Table S3. Summary of mass changes found across varying thermal treatments of ZIF-62.

Sample	Mass (mg) [b]							Mass loss (%)	
(t_m,t_g) [a]	Initial	Solvent Loss	Heating	T_m Hold	Cooling	Heating	T_g Hold	Solvent	Framework
(0,0)	9.60	8.57	8.55	-	8.62	8.78	-	10.7%	-2.5%
(10,0)	9.80	8.67	8.63	8.63	8.60	8.56	-	11.5%	1.3%
(120,0)	11.50	10.14	10.14	10.15	10.13	10.15	-	11.8%	-0.1%
(0,30)	9.90	8.62	8.61	-	8.59	8.59	8.58	12.9%	0.4%
(0,300)	10.00	8.75	8.75	-	8.77	8.78	8.64	12.5%	1.2%
(120,300)	11.20	9.83	9.83	9.99	10.02	10.08	10.18	12.2%	-3.5%
							Average	12.0%	-0.5%

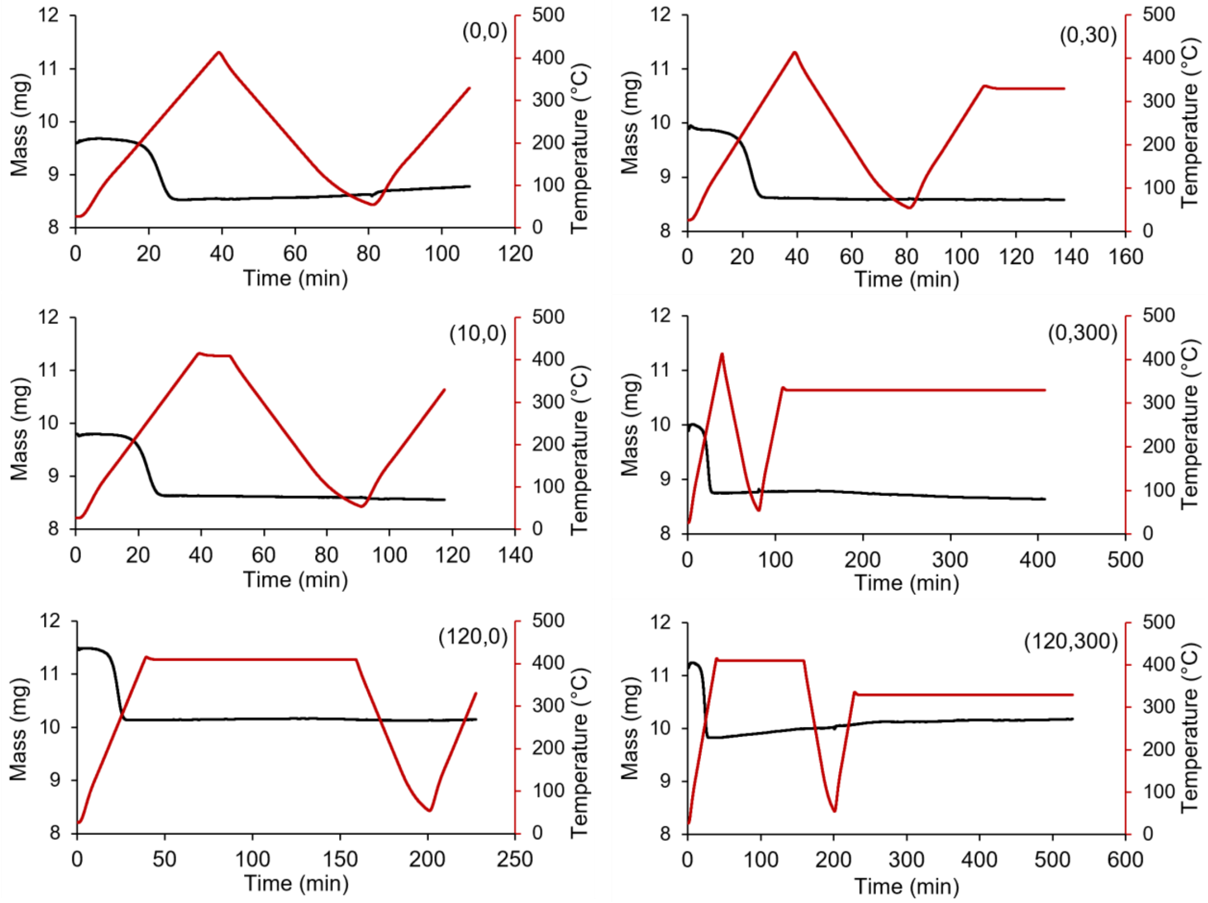

Figure S5. Thermographic analysis profiles of ZIF-62 undergoing thermal treatment into a_gZIF-62. Mass loss onset consistent with the release of DMF (boiling point: 153°C). Mass loss from sample decomposition not seen.

Thermogravimetric analysis combined with differential scanning calorimetry were used to confirm that the solvent loss and melting point of these samples were consistent with previous literature. Indeed, the ZIF-62 produced in this work following literature procedures and matched through PXRD, PALS, and TGA did show the same onset of melting point as previously reported for ZIF-62 (398-410 °C).

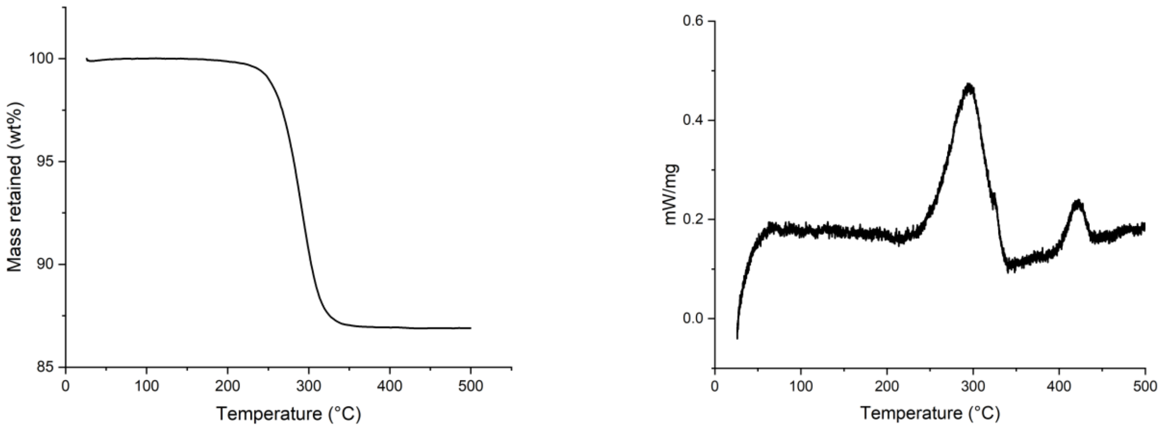

Figure S6. Left: Thermographic analysis profiles of ZIF-62 prepared in this work showing relative mass loss with temperature at a heating rate of 10 °C/min. Right: Differential scanning calorimetry at a heating rate of 10 °C/min of as-prepared ZIF-62 in this work showing the solvent removal at 243 °C and melting at 398-410 °C.

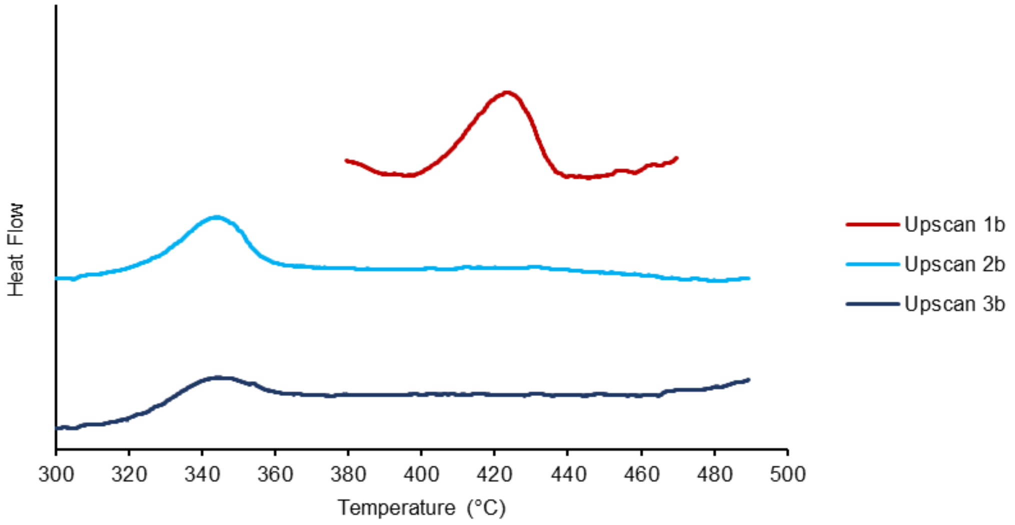

Figure S7. DSC upscan curves of ZIF-62 glass sample (b) during an initial upscan (1b), and repeated upscans (2b, 3b). 1b shows melting occurring at 409.21 °C Repeated scans 2b and 3b show the glass form ZIF-62 transitioning at 328.49 °C and 327.79 °C respectively. Heat flow has been manipulated for improved comparison of samples.

PALS

Having been evacuated under a 1 Pa active vacuum for 62 hrs, and melted following the previously outlined procedures, positron annihilation lifetime spectroscopy was used to identify the changes in porosity of ZIF-62 and a_gZIF-62 caused by differing isothermal treatments. PALS reveals information on the frameworks' pores sizes, along with relative quantities of those pores, without causing any deformation.[7] Following melting, a_gZIF-62 had decreased apertures' diameters and increased cage diameters compared to ZIF-62. The variation between glasses was significant but showed no clear trends with the isothermal treatments.

Table S4. Results and uncertainties of PALS data via a four-component fit model, as well as calculated fractional free volumes for ZIF-62 and subsequent variations of a_gZIF-62

	Pore Diameter (Å)				Intensity (%)				Fractional Free Volume (%)					
Sample ID	D3	±	D4	±	I3	±	I4	±	FFV3	±	FFV4	±	FFVT	±
ZIF-62	3.6	0.1	5.76	0.01	7.8	0.3	19.0	0.3	0.34	0.03	3.44	0.06	3.78	0.07
(0, 0)	3.4	0.1	6.57	0.02	7.1	0.5	19.9	0.1	0.26	0.03	5.32	0.05	5.57	0.06
(10, 0)	3.4	0.2	6.58	0.03	7.2	0.6	19.9	0.3	0.27	0.04	5.4	0.1	5.6	0.1
(20, 0)	3.5	0.1	6.58	0.03	7.0	0.3	19.9	0.3	0.27	0.03	5.33	0.09	5.6	0.1
(30, 0)	3.4	0.1	6.57	0.04	6.9	0.6	19.9	0.3	0.25	0.03	5.3	0.1	5.6	0.1
(60, 0)	3.2	0.1	6.54	0.02	7.4	0.4	20.1	0.2	0.23	0.03	5.30	0.07	5.54	0.08
(120, 0)	3.4	0.2	6.54	0.02	6.8	0.5	20.0	0.2	0.26	0.06	5.26	0.08	5.5	0.1
(0, 30)	3.5	0.2	6.48	0.03	6.6	0.4	19.8	0.5	0.27	0.06	5.1	0.1	5.4	0.2
(0, 60)	3.5	0.2	6.49	0.02	6.0	0.6	20.4	0.2	0.25	0.04	5.25	0.07	5.49	0.08
(0, 300)	3.3	0.2	6.48	0.03	6.7	0.3	20.2	0.3	0.23	0.04	5.2	0.1	5.4	0.1
(120, 300)	3.7	0.2	6.52	0.02	6.7	0.7	19.0	0.2	0.33	0.06	4.97	0.08	5.3	0.1

Fractional free volume calculations are based on a cylindrical pore model, given by equation **1** below. [5f, 8]

$$FFV = C\frac{4}{3}\pi r^3 I \qquad 1$$

Where C is the constant 0.0018 $Å^3$, r (Å) is the pore radii and I (%) is the intensity found via PALS.

Adsorption

Adsorption/desorption isotherms were collected using CO_2, N_2, and propene to link the porosity found via PALS to the potential real applications of the ZIF-62 glasses. The adsorption capacity of these gases was linked not just to the porosity of the samples, but also the affinity to the frameworks (heat of adsorption). In ZIF-62, CO_2 and propene had similar adsorption capacities at standard temperature/pressure, however following glass transformation propene was more greatly affected, resulting in a higher relative loss of capacity. N_2 had a low affinity to the framework, though still exhibited a capacity reduction in a_gZIF-62.

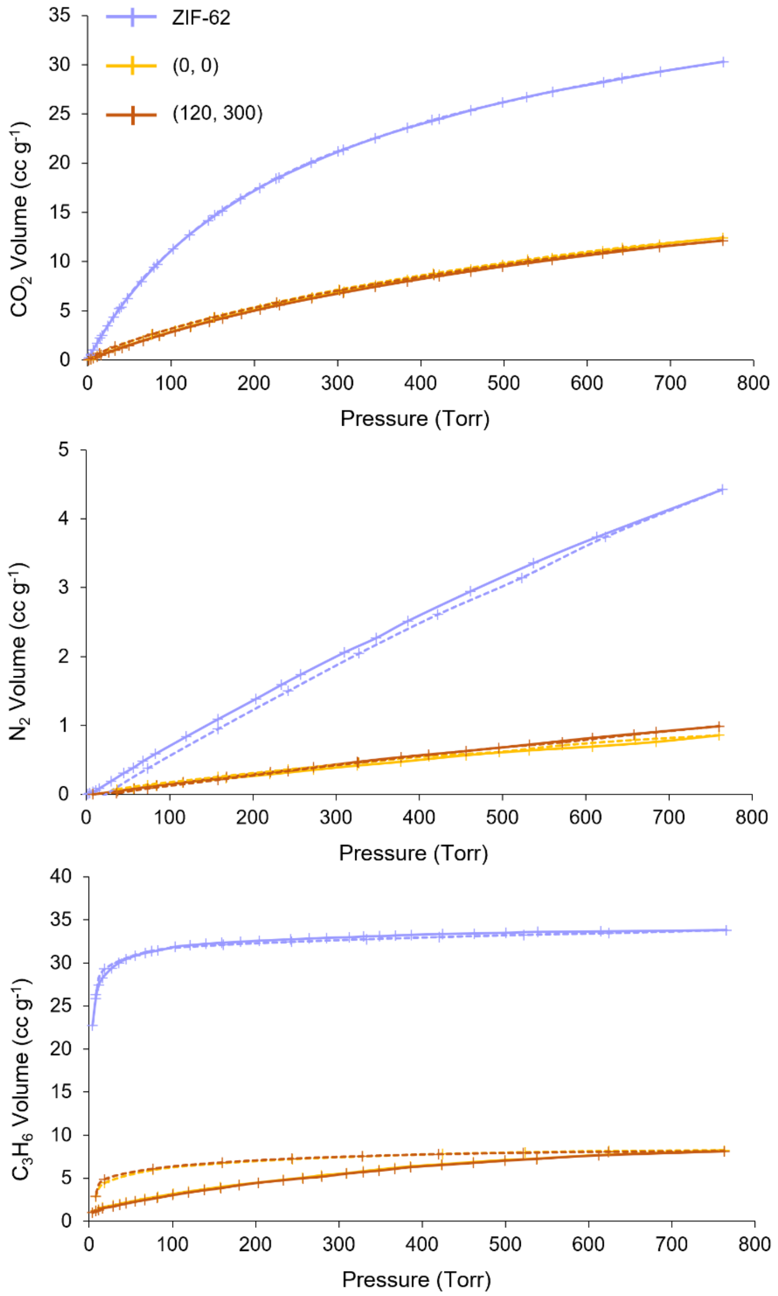

Figure S8. Adsorption (solid lines) and desorption (dashed lines) isotherms of CO_2, N_2, and C_3H_6, for ZIF-62, (0, 0) and (120, 300) from vacuum to standard pressure at standard temperature.

Morphological and Optical Changes

Further physical characteristics brought on by the isothermal treatments were tracked. Optical changes, being how much the glasses darkened gave information to the level of decomposition occurring within the framework, which increased with treatment length.[4] Morphological changes between ZIF-62 and a_gZIF-62 variants were tracked to identify if melting behaviour would impact the formation of thin films or other designed structures. Longer thermal treatments showed larger morphological changes, including improved coalescence of adjacent grains, as necessary for thin film production. Whereas short treatments on macro-crystals retained more characteristics of the initial ZIF-62 grain.

Table S5. Bulk a_gZIF-62 following various thermal treatments. Values of the glasses show a decrease with longer thermal treatments.

Sample	(0,0)	(10,0)	(20,0)	(30,0)	(60,0)	(120,0)	(0,30)	(0,60)	(0,300)	(120,300)
Image[a]	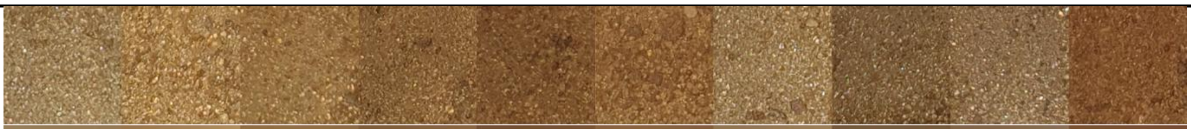									
Hue[b]	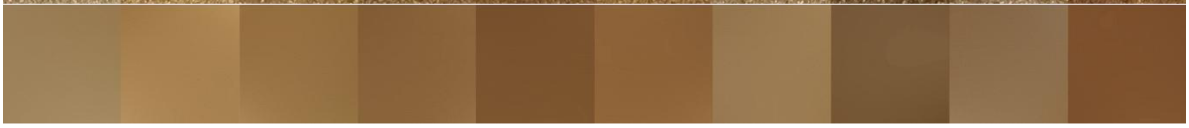									
Value[c]	127	127	111	97	86	100	122	91	111	86

[a] Images collected under artificial light for consistent comparison. [b] Gaussian average hue for each set of bulk sample. [c] Value, also known as luminosity or lightness were collected via the hues RGB code. The formula 1/3(R+G+B) produces values on a scale from 255 (white) to 0 (black)[9]

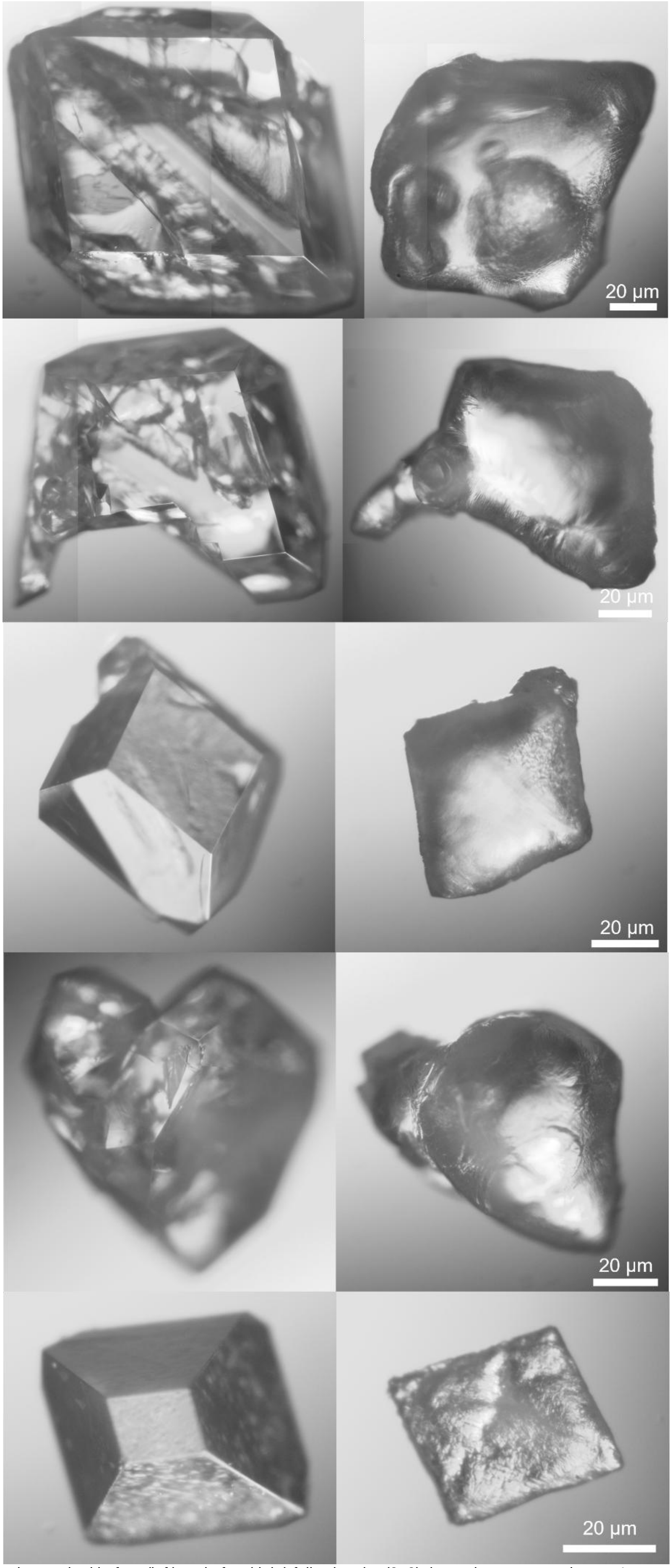

Figure S9. Individual ZIF-62 grains tracked before (left) and after (right) following the (0, 0) thermal treatment, taken at 4x magnification.

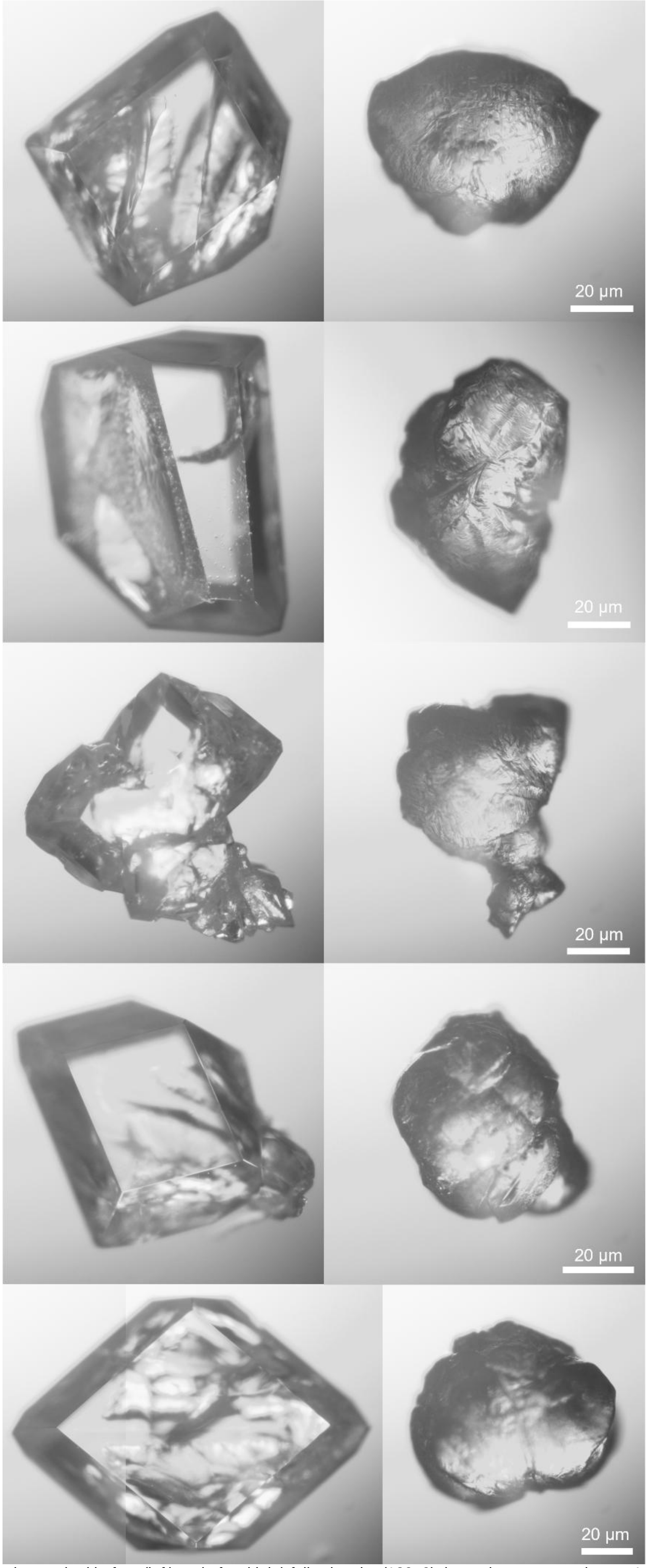

Figure S10. Individual ZIF-62 grains tracked before (left) and after (right) following the (120, 0) thermal treatment, taken at 4x magnification.

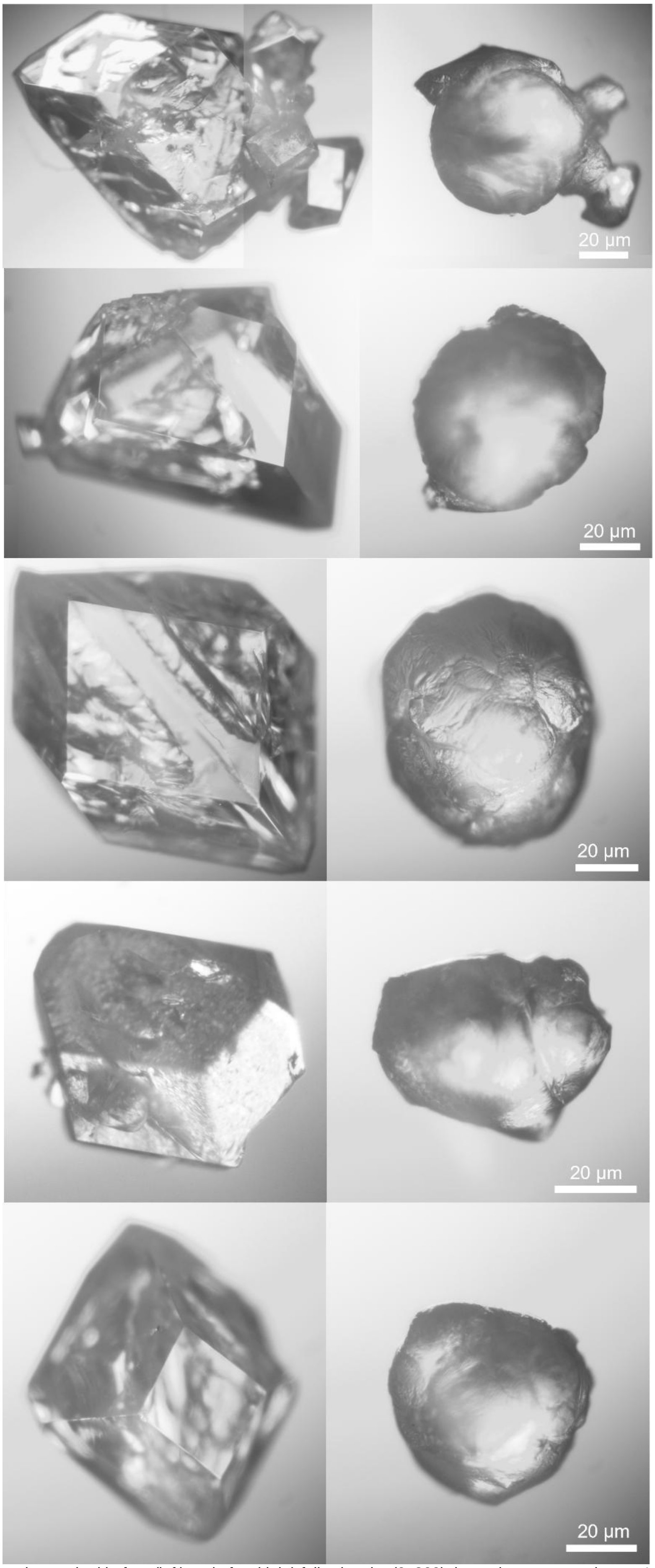

Figure S11. Individual ZIF-62 grains tracked before (left) and after (right) following the (0, 300) thermal treatment, taken at 4x magnification.

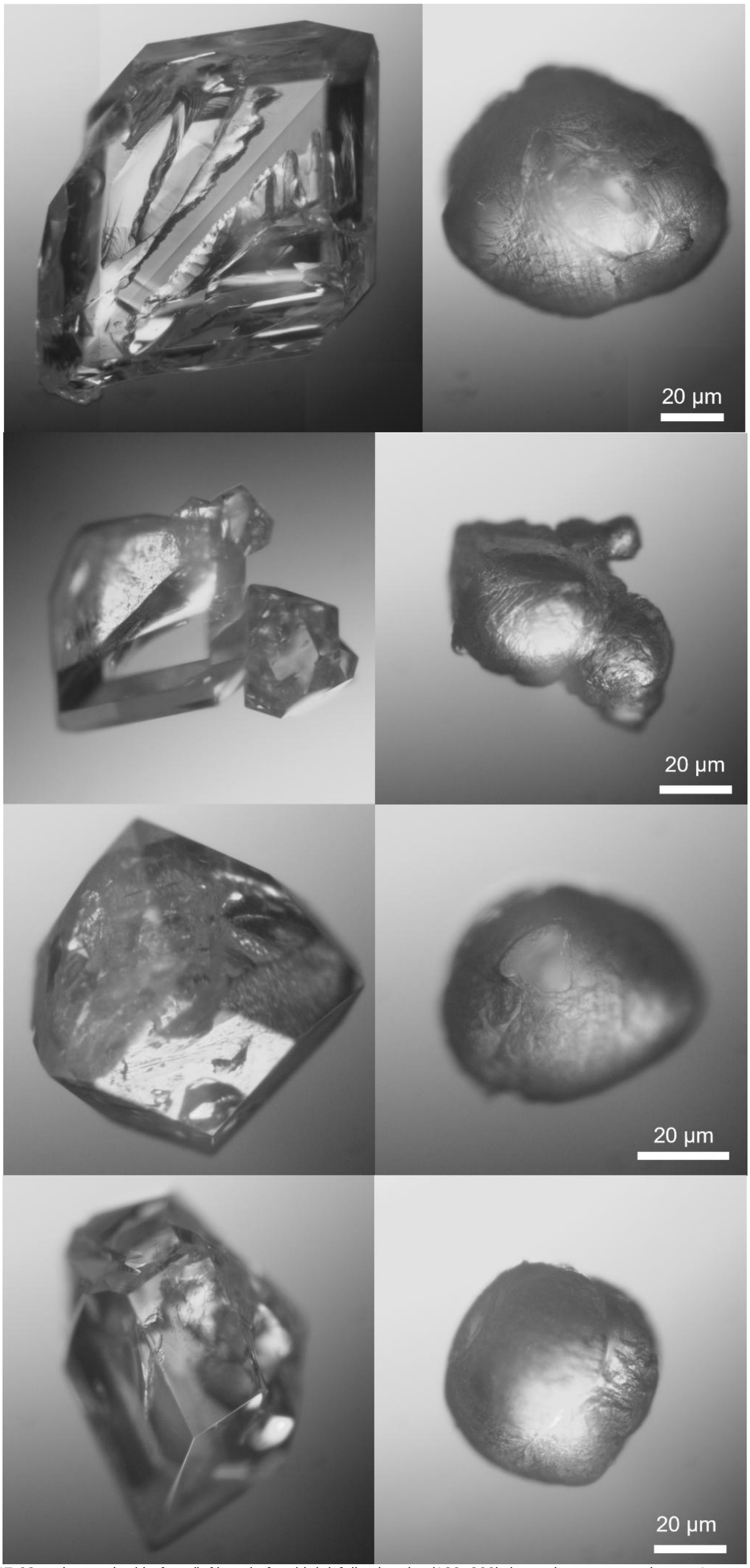

Figure S12. Individual ZIF-62 grains tracked before (left) and after (right) following the (120, 300) thermal treatment, taken at 4x magnification.

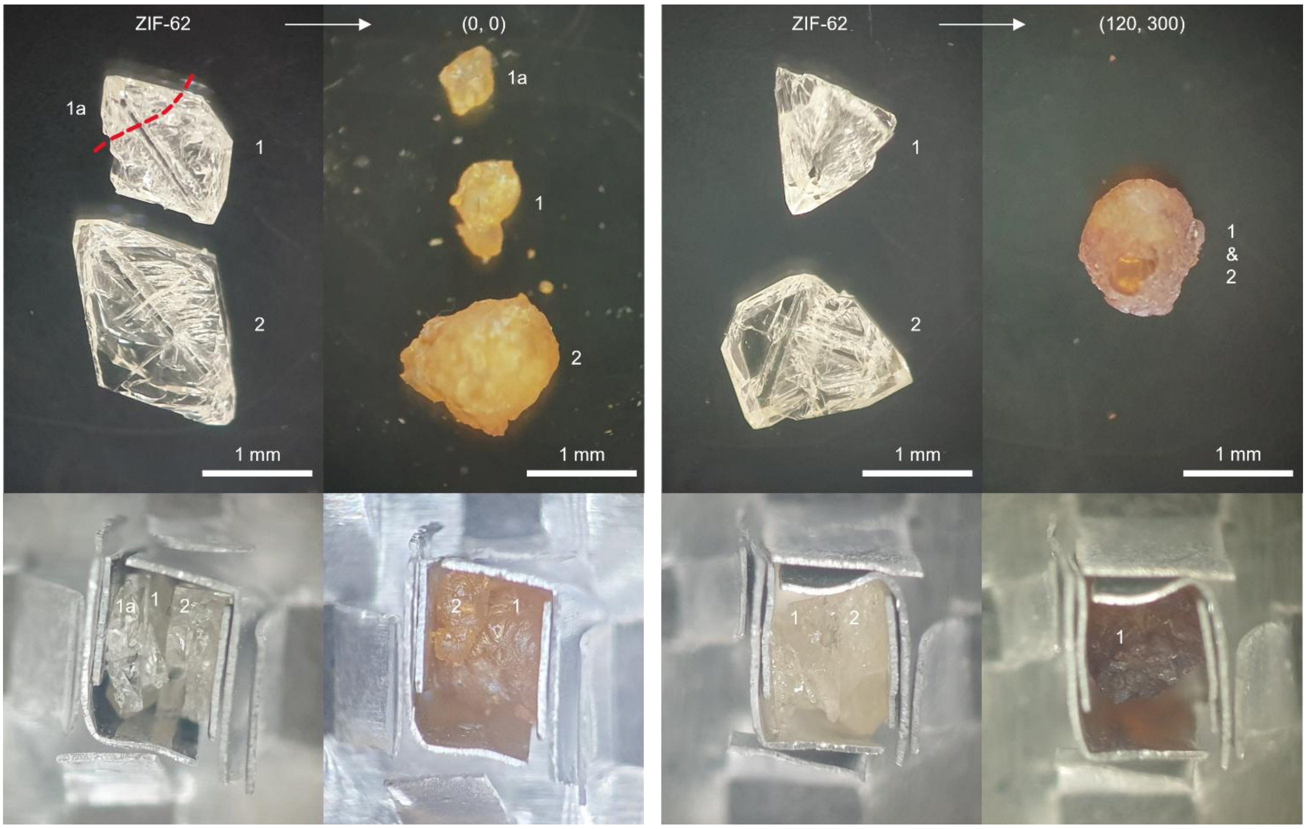

Figure S13. Effect of isothermal treatments on paired ZIF-62 grains. Under a short melting period, each grain remained separate, whilst under a long melting period, the grains fully coalesced showing no sign of the initial grain boundaries. Note segment 1a on the left detached prior to melting.

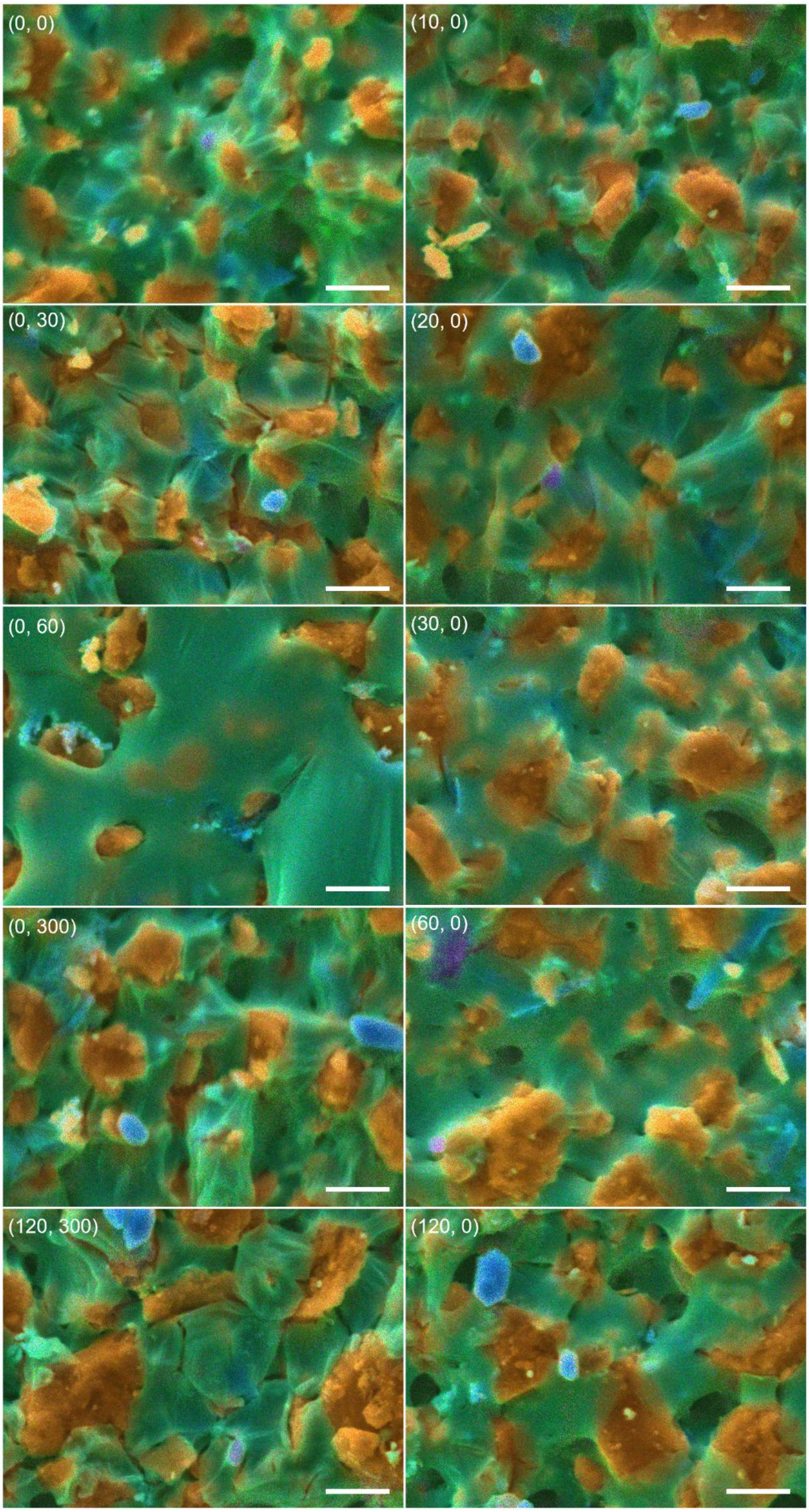

Figure S14. Microscale melting of ZIF-62 onto an alumina thin sheet, imaged via SEM. EDS was used to identify the elemental composition and has been colour coded as aluminium in orange, zinc in green and contaminant elements (Silicon, Calcium) in dark blue. Scale bars are 10 µm.

^{1}H NMR and infrared characterization

The ^{1}H NMR spectra of the pristine (9.1 mg; observed ligand ratio 6 imidazole: 1 benzimidazole) and melted ZIF-62 (120, 300) (8.7 mg; observed ligand ratio 7 imidazole: 1 benzimidazole) were collected to confirm the original ligand ratio of the ZIF-62 and to confirm observations previously reported in the literature where the ligand ratio is observed to change upon melting.[5f] NMR solutions were prepared by digesting the ZIF-62 samples in 0.1 mL of D_2O, followed by addition of 0.6 mL of d_6-DMSO.[5f] We also collected the supersaturated ^{1}H NMR spectrum of melted ZIF-62 in an attempt to identify the byproducts responsible for the observed darkening of the ZIF-62 samples (115 mg/mL). As expected, no peaks with measurable integration were observed, indicating that the concentration of the coloured molecules is <0.1 wt%.

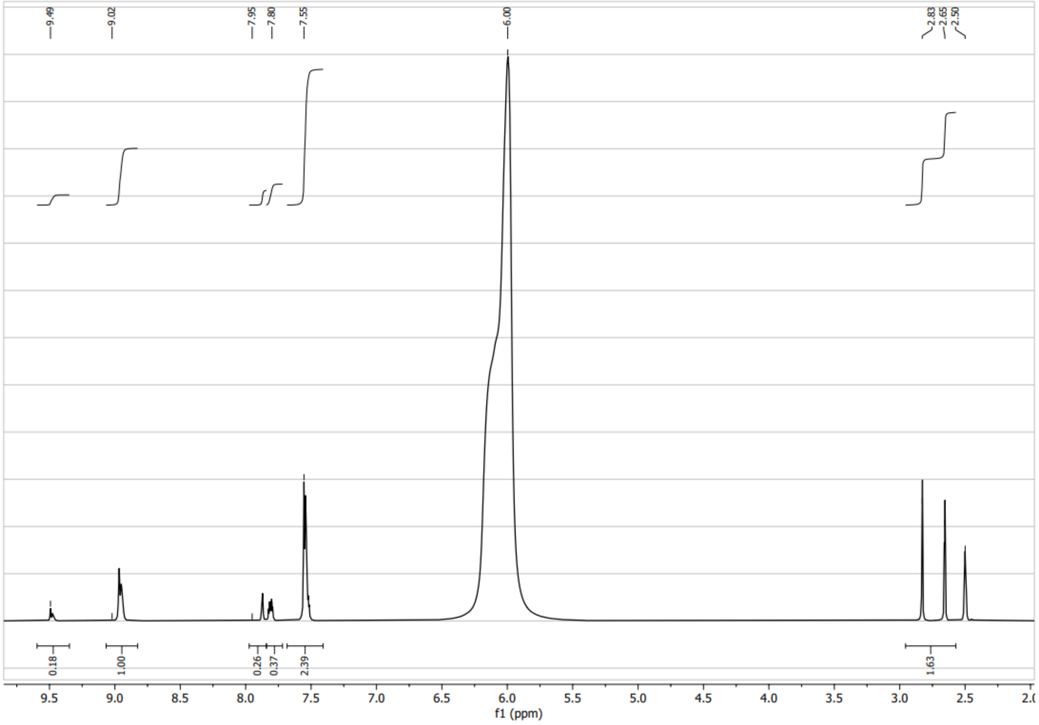

Figure S15. ^{1}H NMR spectrum of as-prepared ZIF-62, 9.1 mg in 0.1 mL DCl and 0.6 mL d_6-DMSO showing a 6 imidazole:1 benzimidazole ligand ratio.

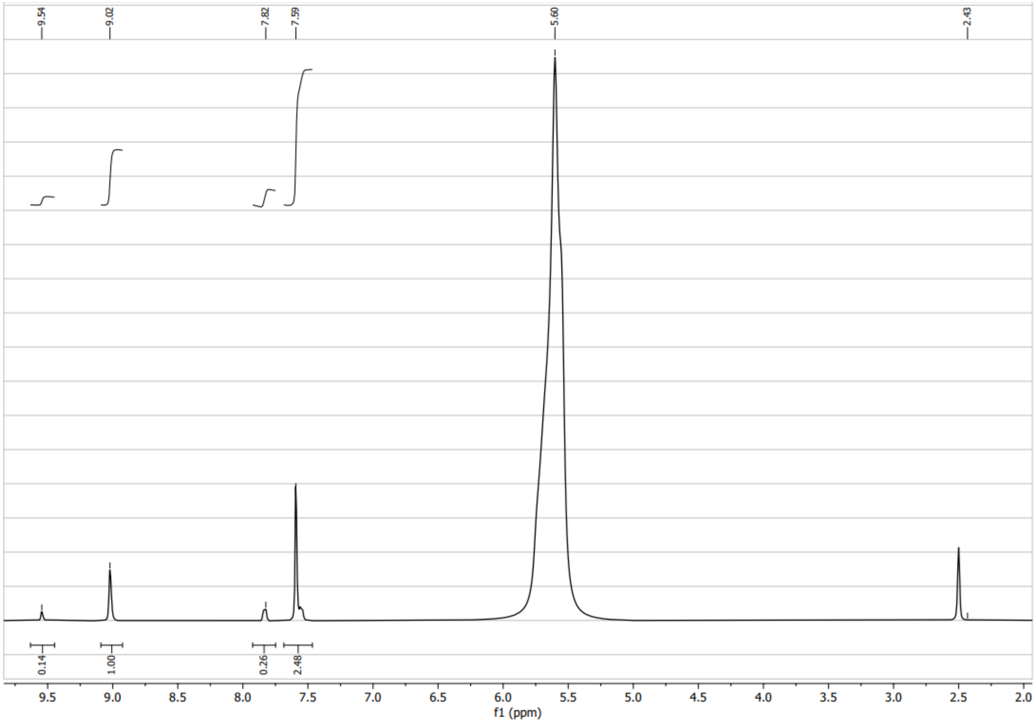

Figure S16. ^{1}H NMR spectrum of melted ZIF-62 (120, 300), 8.7.1 mg in 0.1 mL DCl and 0.6 mL d_6-DMSO showing a 6 imidazole:1 benzimidazole ligand ratio.

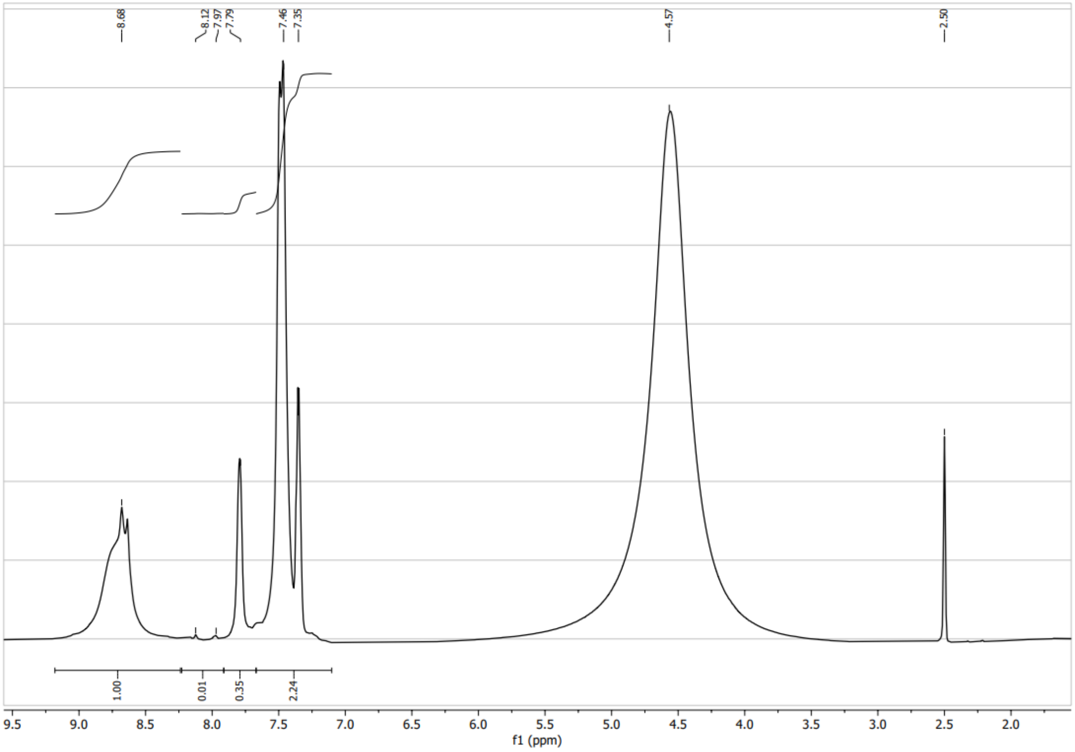

Figure S17. ^{1}H NMR spectrum of melted ZIF-62, 81 mg in 0.1 mL DCl and 0.6 mL d_6-DMSO in an attempt to notice and identify the coloured species responsible for the colouration of the melted glass samples.

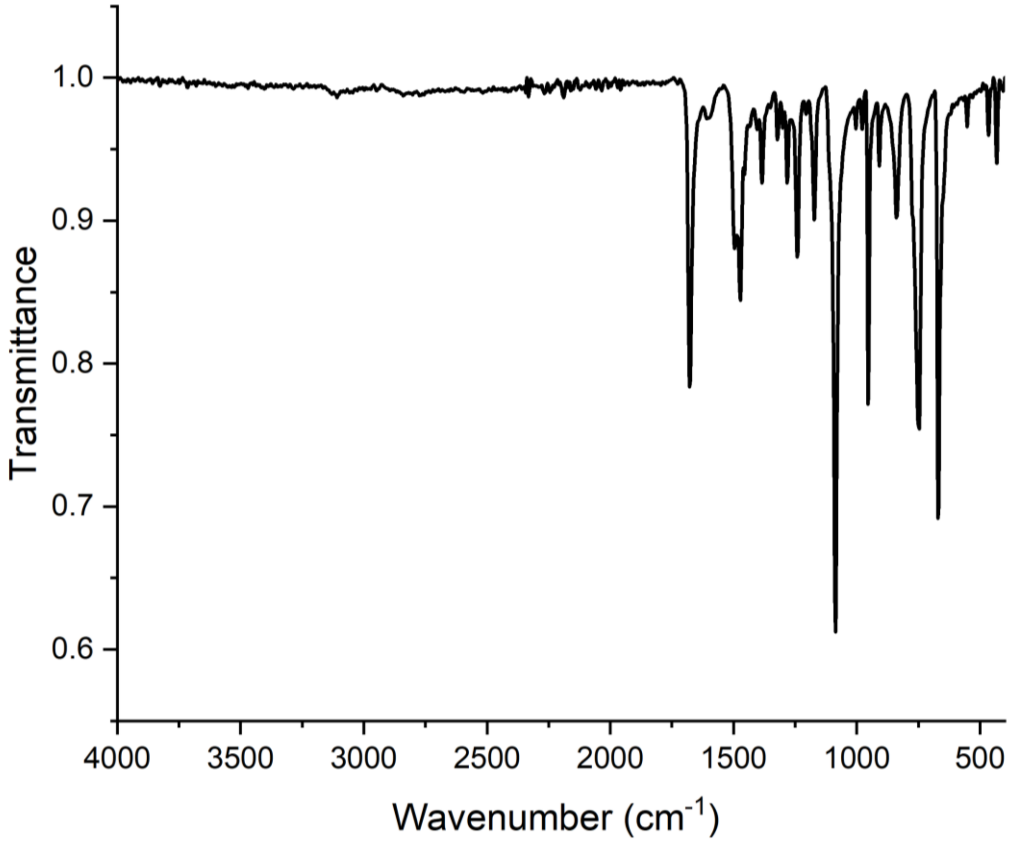

Figure S18. Infrared spectrum recorded for as-prepared ZIF-62.

Supporting Information

References

[1] D. M. Stone, S. E. Morgan, M. O. Abdelmigeed, J. Nguyen, T. D. Bennett, G. N. Parsons, M. G. Cowan, *Small* **2024**, *n/a*, 2307202.

[2] a) L. Frentzel-Beyme, M. Kloß, R. Pallach, S. Salamon, H. Moldenhauer, J. Landers, H. Wende, J. Debus, S. Henke, *Journal of Materials Chemistry A* **2019**, *7*, 985-990; b) L. Frentzel-Beyme, M. Kloß, P. Kolodzeiski, R. Pallach, S. Henke, *Journal of the American Chemical Society* **2019**, *141*, 12362-12371.

[3] J. Kansy, *Nuclear Instruments and Methods in Physics Research Section A: Accelerators, Spectrometers, Detectors and Associated Equipment* **1996**, *374*, 235-244.

[4] a) S. J. Tao, *The Journal of Chemical Physics* **1972**, *56*, 5499-5510; b) M. Eldrup, D. Lightbody, J. N. Sherwood, *Chemical Physics* **1981**, *63*, 51-58.

[5] a) Z. Zhao, L. Ding, A. Mundstock, O. Stölting, S. Polarz, H. Wang, A. Feldhoff, *Journal of Membrane Science* **2024**, *700*, 122677; b) D. Li, Z. Yang, L. Yang, C. Ma, M. Ye, Y. Sun, Z. Qiao, A. Chen, *Journal of Membrane Science* **2024**, *695*, 122492; c) A. Acharya, B. Chanda, M. Saminathan, S. Perumal, K. Jayanthi, K. Annapurna, N. M. A. Krishnan, B. Gahtori, M. K. Naskar, S. Ghosh, A. R. Allu, S. K. Mishra, *Journal of Non-Crystalline Solids* **2024**, *627*, 122816; d) Y. Feng, W. Yan, Z. Kang, X. Zou, W. Fan, Y. Jiang, L. Fan, R. Wang, D. Sun, *Chemical Engineering Journal* **2023**, *465*, 142873; e) T. D. Bennett, Y. Yue, P. Li, A. Qiao, H. Tao, N. G. Greaves, T. Richards, G. I. Lampronti, S. A. T. Redfern, F. Blanc, O. K. Farha, J. T. Hupp, A. K. Cheetham, D. A. Keen, *Journal of the American Chemical Society* **2016**, *138*, 3484-3492; f) A. Qiao, T. D. Bennett, H. Tao, A. Krajnc, G. Mali, C. M. Doherty, A. W. Thornton, J. C. Mauro, G. N. Greaves, Y. Yue, *Science Advances* **2018**, *4*, eaao6827; g) C. Zhou, M. Stepniewska, L. Longley, C. W. Ashling, P. A. Chater, D. A. Keen, T. D. Bennett, Y. Yue, *Physical Chemistry Chemical Physics* **2018**, *20*, 18291-18296; h) S. Li, R. Limbach, L. Longley, A. A. Shirzadi, J. C. Walmsley, D. N. Johnstone, P. A. Midgley, L. Wondraczek, T. D. Bennett, *Journal of the American Chemical Society* **2019**, *141*, 1027-1034; i) S. Li, S. Yu, S. M. Collins, D. N. Johnstone, C. W. Ashling, A. F. Sapnik, P. A. Chater, D. S. Keeble, L. N. McHugh, P. A. Midgley, D. A. Keen, T. D. Bennett, *Chemical Science* **2020**, *11*, 9910-9918; j) A. Qiao, H. Tao, M. P. Carson, S. W. Aldrich, L. M. Thirion, T. D. Bennett, J. C. Mauro, Y. Yue, *Opt. Lett.* **2019**, *44*, 1623-1625; k) M. Stepniewska, K. Januchta, C. Zhou, A. Qiao, M. M. Smedskjaer, Y. Yue, *Proceedings of the National Academy of Sciences* **2020**, *117*, 10149-10154; l) L. Longley, S. M. Collins, S. Li, G. J. Smales, I. Erucar, A. Qiao, J. Hou, C. M. Doherty, A. W. Thornton, A. J. Hill, X. Yu, N. J. Terrill, A. J. Smith, S. M. Cohen, P. A. Midgley, D. A. Keen, S. G. Telfer, T. D. Bennett, *Chemical Science* **2019**, *10*, 3592-3601; m) M. Stepniewska, M. B. Østergaard, C. Zhou, Y. Yue, *Journal of Non-Crystalline Solids* **2020**, *530*, 119806; n) Y. Wang, H. Jin, Q. Ma, K. Mo, H. Mao, A. Feldhoff, X. Cao, Y. Li, F. Pan, Z. Jiang, *Angewandte Chemie International Edition* **2020**, *59*, 4365-4369; o) R. Lin, J. Hou, M. Li, Z. Wang, L. Ge, S. Li, S. Smart, Z. Zhu, T. D. Bennett, V. Chen, *Chemical Communications* **2020**, *56*, 3609-3612; p) M. Mubashir, L. F. Dumée, Y. Y. Fong, N. Jusoh, J. Lukose, W. S. Chai, P. L. Show, *Journal of Hazardous Materials* **2021**, *415*, 125639; q) T. To, S. Sørensen, M. Stepniewska, A. Qiao, L. Jensen, M. Bauchy, Y. Yue, M. Smedskjaer, *Nature Communications* **2020**, *2593*, 1234567890; r) V. Nozari, C. Calahoo, L. Longley, T. D. Bennett, L. Wondraczek, *The Journal of Chemical Physics* **2020**, *153*; s) C. W. Ashling, L. K. Macreadie, T. J. F. Southern, Y. Zhang, L. N. McHugh, R. C. Evans, S. Kaskel, S. G. Telfer, T. D. Bennett, *Journal of Materials Chemistry A* **2021**, *9*, 8386-8393; t) L. Frentzel-Beyme, P. Kolodzeiski, J.-B. Weiß, A. Schneemann, S. Henke, *Nature Communications* **2022**, *13*, 7750; u) Y. Zhang, Y. Wang, H. Xia, P. Gao, Y. Cao, H. Jin, Y. Li, *Chemical Communications* **2022**, *58*, 9548-9551; v) Z. Yang, Y. Belmabkhout, L. N. McHugh, D. Ao, Y. Sun, S. Li, Z. Qiao, T. D. Bennett, M. D. Guiver, C. Zhong, *Nature Materials* **2023**, *22*, 888-894.

[6] M. F. Thorne, M. L. R. Gómez, A. M. Bumstead, S. Li, T. D. Bennett, *Green Chemistry* **2020**, *22*, 2505-2512.

[7] a) T. Stassin, R. Verbeke, A. J. Cruz, S. Rodríguez-Hermida, I. Stassen, J. Marreiros, M. Krishtab, M. Dickmann, W. Egger, I. F. J. Vankelecom, S. Furukawa, D. De Vos, D. Grosso, M. Thommes, R. Ameloot, *Advanced Materials* **2021**, *33*, 2006993; b) D. W. Siderius, N. A. Mahynski, V. K. Shen, *Adsorption (Boston)* **2017**, *23*, 593-602.

[8] a) X. Hong, Y. C. Jean, H. Yang, S. S. Jordan, W. J. Koros, *Macromolecules* **1996**, *29*, 7859-7864; b) A. W. Thornton, K. E. Jelfs, K. Konstas, C. M. Doherty, A. J. Hill, A. K. Cheetham, T. D. Bennett, *Chemical Communications* **2016**, *52*, 3750-3753.

[9] A. Hanbury, *Pattern Recognition Letters* **2008**, *29*, 494-500.
